# Role of Viral Envelope Proteins in Determining Susceptibility of Viruses to IFITM Proteins

**DOI:** 10.3390/v16020254

**Published:** 2024-02-05

**Authors:** Thomas Marceau, Martine Braibant

**Affiliations:** Inserm U1259 MAVIVH, Université de Tours, 37032 Tours, France

**Keywords:** IFITM, virus, envelope, restriction, escape, susceptibility

## Abstract

Interferon-induced transmembrane proteins (IFITMs) are a family of proteins which inhibit infections of various enveloped viruses. While their general mechanism of inhibition seems to be non-specific, involving the tightening of membrane structures to prevent fusion between the viral envelope and cell membrane, numerous studies have underscored the importance of viral envelope proteins in determining the susceptibility of viruses to IFITMs. Mutations in envelope proteins may lead to viral escape from direct interaction with IFITM proteins or result in indirect resistance by modifying the viral entry pathway, allowing the virus to modulate its exposure to IFITMs. In a broader context, the nature of viral envelope proteins and their interaction with IFITMs can play a crucial role in the context of adaptive immunity, leading to viral envelope proteins that are more susceptible to antibody neutralization. The precise mechanisms underlying these observations remain unclear, and further studies in this field could contribute to a better understanding of how IFITMs control viral infections.

## 1. Introduction

### 1.1. IFITM Discovery

The interferon-induced transmembrane (IFITM) protein family was discovered by Friedman et al. in 1984 during the analysis of mRNAs induced after interferon (IFN) treatment of neuroblastoma cells [1]. The gene locus corresponding to this protein family was initially named 1-8 and is highly induced by both type I and type II IFN [2]. Three genes, 9-27, 1-8D, and 1-8U, encoding functional members of this protein family, were later isolated by Lewin et al. on a single genomic DNA fragment of less than 18 kb [3]. They harbor interferon-stimulated response elements (ISRE) in their 5*’* flank, and cDNAs of the three genes share more than 90% identity over 70% of the coding sequence. These genes were then referred to as *ifitm1* (9-27), *ifitm2* (1-8D), and *ifitm3* (1-8U), with two new members, *ifitm5* and *ifitm10*, joining this family. All five genes are located on human chromosome 11. However, in contrast to *ifitm1*, *ifitm2*, and *ifitm3*, *ifitm5* and *ifitm10* are insensitive to IFNs. While IFITM1, IFITM2, and IFITM3 proteins have been widely studied for their broad antiviral activity, little is known about the roles of IFITM5 and IFITM10, which lack antiviral function.

### 1.2. IFITM1, IFITM2, and IFITM3 Structure and Cellular Localization

**Figure 1:**
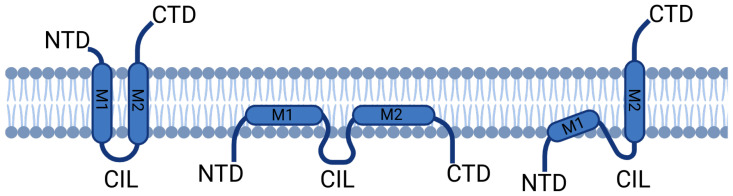
Schematic representation of proposed models of IFITM proteins’ structure. The overall structure contains two membrane domains (M1 and M2) linked by a conserved intracellular loop (CIL) and flanked by a N-terminal domain (NTD) and a C-terminal domain (CTD). In the first model (left), there are two transmembrane domains, and the NTD and CTD are exposed extracellularly. In the second model (middle), both the NTD and CTD are exposed intracellularly, and the M1 and M2 domains do not span the membrane. The third model, which is currently the most accepted, proposes an intracellular NTD and an extracellular CTD, with the M1 not spanning the membrane, but the M2 being transmembrane.

As described above, the genomic sequences of *ifitm1*, *ifitm2*, and *ifitm3* are quite similar. At the amino acid level, IFITM3 and IFITM2 share 90.2% sequence homology, and IFITM3 and IFITM1 share 73.7% sequence similarity. All three contain two hydrophobic putative membrane-interacting domains [3], though their topologies remain ambiguous. Three models have been suggested, all composed of the same five domains: the N-terminal domain (NTD), the membrane domain I (M1), the conserved intracellular loop (CIL), the membrane domain II (M2), and the C-terminal domain (CTD) (Figure 1).

It was initially proposed that the NTD and CTD are extracellularly located, connected by two transmembrane domains (M1 and M2) and the CIL (Figure 1 left). In support of this prediction, the NTD and CTD of IFITM3 have been detected on cell surfaces by immunostaining [4,5]. However, this first model was subsequently challenged by studies of post-translational modifications of IFITM3 (S-palmitoylation, ubiquitination, and phosphorylation) which show that the NTD and CIL are modified by cytoplasmic enzymes [6,7]. A second model was proposed in which the NTD, CTD, and CIL are in the intracellular part, suggesting that the domains M1 and M2 do not traverse the membrane (Figure 1 middle). Later, Bailey et al. proposed a type II transmembrane protein model for IFITM3. In this third model, the NTD and CIL are intracellular, implying that the M1 domain does not span the membrane while the CTD is extracellular, thus requiring that the M2 domain traverses the membrane (Figure 1 right). This model was based on a comparison of surface exposure of the NTD and CTD by cytometry and immunofluorescence as well as the analysis of their glycosylation and lysosomal degradation [8]. This model was further supported using combined approaches of electron paramagnetic resonance (EPR) and nuclear magnetic resonance (NMR), suggesting the presence of a single transmembrane helix [9]. This last model is currently the most commonly accepted.

Even though the protein structure is conserved within this family, there are unique characteristics of each member. IFITM1 is shorter than IFITM2 and IFITM3, with IFITM1 having a shorter NTD but a longer CTD [10]. The NTD and CTD are the domains that exhibit the most diversity among the different IFITMs. IFITM proteins can be found in both the plasma membrane and endolysosomal membranes. The specific type of membrane where each IFITM is located varies. IFITM1 is primarily located at the plasma membrane and early endosomes while IFITM2 and IFITM3 are predominantly found in late endosomes and lysosomes [11]. For IFITM3, it has been shown that the NTD plays a crucial role in its subcellular localization, with the Y20 amino acid being a key residue of the YxxΦ motif required for correct localization in endosomes and its antiviral action [7]. These proteins can still be observed in lower proportion on the plasma membrane.

### 1.3. IFITM1, IFITM2, and IFITM3 as Antiviral Factors

Extensive research on IFITM1, IFITM2, and IFITM3 is primarily driven by their remarkable capacity to restrict various viruses, including human immunodeficiency virus type 1 (HIV-1), influenza A virus (IAV), West Nile virus, Dengue virus, Zika virus, Ebola virus, Rift Valley Fever virus, alphaviruses, reoviruses, Kaposi sarcoma-associated herpesvirus, Chikungunya virus, Mayaro virus, and severe acute respiratory syndrome coronavirus (SARS-CoV) [4,12,13,14,15,16,17,18,19,20,21,22]. Compared with IFITM1 and IFITM2, IFITM3 is considered to be the most potent antiviral isoform [23].

In the case of HIV-1 restriction, it has been demonstrated that target cells expressing IFITM2 and IFITM3 on their membranes can protect themselves from infection by inhibiting the fusion between the viral envelope and the cellular membrane [24]. Subsequently, it was found that IFITM proteins, particularly IFITM3, can be incorporated into virions during budding, leading to the generation of less infectious viral particles [25,26]. This phenomenon is observed in a wide range of viruses [27], indicating that IFITM proteins interfere with virus replication at two stages of their life cycle: in target cells by retaining viral particles in endosomes and in producer cells by producing virions with reduced infectivity.

It is now well established that IFITM proteins exert their antiviral action during the fusion process. However, the exact molecular mechanisms underlying this inhibition are not yet fully elucidated. Several non-mutually exclusive models have been proposed based on studies of different virus families. The first model suggests that IFITM proteins rigidify the cellular membrane, leading to a decrease in the fusion process [28,29,30]. Another model suggests that a modification of the endosomal membrane composition with cholesterol accumulation could contribute to blocking this fusion process [31]. However, the second model is still debated, as several independent research teams have shown that IFITM3 restriction is independent of cholesterol excess in endosomes [22,32]. While the first two models can explain the protection of target cells from viral infection, their contribution to the inhibitory effect of IFITM proteins due to their incorporation into virions remains unknown. Finally, some studies on retroviruses have indicated a decrease in the incorporation of viral envelope proteins into virions after overexpression of IFITM proteins in virion-producing cells [33,34]. This could explain the reduction in the infectivity of viral particles in the presence of IFITM proteins. Nevertheless, this model remains controversial, particularly under conditions where the expression levels of IFITM proteins are similar to those observed in primary cells stimulated by type I IFN [25,26,35,36].

Taken together, these observations show that IFITM proteins are potent inhibitors of various enveloped viruses, blocking virus entry and leading to the budding of less infectious virions. Interestingly, we will see that the antiviral effects of these proteins are tightly linked to the nature of viral envelopes and to the specificities of viral replication cycles.

## 2. Diversity of Viral Envelopes Proteins and IFITMs

### 2.1. Viral Resistance to IFITMs Depends on Envelope Surface Proteins

Early studies suggested that viruses could be either sensitive or resistant to IFITM proteins, and this trait was initially believed to be inherent to each viral species. However, it soon became clear that, at least for some viruses, their sensitivity to IFITMs was strain- or variant-specific [12]. Initial investigations performed on a few laboratory-adapted strains of HIV-1, such as BH1, demonstrated their restriction by IFITMs [24]. Nevertheless, some laboratory-adapted strains like AD8 and primary isolates of HIV-1 that escape the antiviral activity of IFITM proteins were subsequently reported [36,37,38].

Focusing on IFITMs expressed in target cells, the work of Foster et al. revealed that transmitted/founder (T/F) variants of HIV-1, isolated early in infection, exhibited greater resistance to IFITM2 and IFITM3 restriction compared to viral variants isolated during the chronic phase of infection [37]. The observed IFITM phenotypes were transferable to lentiviruses pseudotyped with the respective Env variants, indicating that amino acids changes in the envelope glycoproteins are determinants of IFITM-mediated restriction. When comparing viruses that use different co-receptors (CCR5 or CXCR4), Foster et al. also reported that CCR5-using viruses (R5) tended to be more sensitive to IFITM1 but more resistant to IFITM2/3 than CXCR4-using viruses (X4). This phenotype was attributed to the V3 loop of Env [37]. However, using a large set of HIV-1 strains, including CCR5-, CXCR4- or dual-tropic strains, Yu et al. obtained conflicting results regarding co-receptor usage dependence [39]. More recently, the work of Haider et al. revealed that the cytoplasmic domain of Env is another determinant of susceptibility to IFITM proteins, with its truncation rendering viruses resistant [38].

Similarly, when focusing on the inhibitory activity of IFITMs after incorporation into newly formed viruses, Wang et al. reported that sensitivity to IFITM2/3 is strain-specific [36]. In contrast, virus sensitivity to IFITM1 appears to be consistently low, regardless of the strain. Once again, resistance to IFITM3 action has been attributed to the V3 variable loop of gp120 [36]. More recently, using a broader panel of Env clones originating from laboratory-adapted or primary strains, we confirmed the crucial role played by Env in modulating susceptibility to IFITM3 [35]. By swapping variable domains of Env between sensitive and resistant clones, we demonstrated that both the V3 and V1V2 loops are genetic determinants of resistance to the antiviral activity of IFITM3. However, no link between IFITM3 inhibition of HIV-1 infectivity and HIV-1 coreceptor usage could be observed [35,36]. Using a panel of lentiviruses pseudotyped with Env clones derived from either T/F HIV-1 strains, acute or chronic infections, Beitari et al. also reported that T/F Env clones are relatively resistant to the viral incorporation of IFITM3, but this resistance property diminishes as the infection progresses. This supports the findings by Foster et al. when IFITMs are expressed in target cells [37,40]. One proposed scenario is that adaptive immune pressure and the emergence of neutralizing antibody resistance mutations in HIV-1 Env have led to viral isolates that are more sensitive to IFITM proteins.

Collectively, papers showing the importance of Env in modulating susceptibility of HIV-1 to IFITMs are further supported by the capacity of HIV-1 to overcome IFITM restriction by mutating its *env* gene during prolonged replication in cell culture [33,41]. Interestingly, while most studies were based on infection of target cells by cell-free HIV-1 particles, the work of Yu et al. carried out in the context of co-culture of HIV-1-infected donor cells with target cells to mimic cell-to-cell HIV transmission, also showed the strain specificity of IFITM inhibition [33].

The susceptibility of hepatitis C virus (HCV) to IFITM proteins has also been reported to be envelope-dependent [42]. Using HCV pseudoparticles (HCVpp)-bearing envelopes (E1E2) from patients with acute infection before seroconversion or patients undergoing liver transplantation due to chronic hepatitis C, Wrench et al. compared envelopes’ susceptibility to the presence of IFITM proteins overexpressed in Huh7.5.1 target cells. Results differed to what was observed for HIV-1. Firstly, the antiviral activity did not significantly differ between IFITM1, IFITM2, and IFITM3 proteins. Secondly, HCV variants isolated pre-seroconversion were more sensitive to IFITM proteins than variants isolated from patients during chronic infection, suggesting that IFITM proteins may exert significant selective pressure on HCV during the acute phase of infection, resulting in viral evasion.

The antiviral pressure exerted by IFITM proteins was also observed for SARS-CoV-2. Following the identification of the first SARS-CoV-2 strain circulating in humans in 2020 (Wuhan-Hu-1), dominant variants of concern (VOCs) with an increasing number of mutations in the spike protein emerged. The initial VOC, Alpha, which became the dominant variant in much of Europe and North America in the first months of 2021, was shown to be more resistant to IFITMs than the parental Wuhan-Hu-1 virus, with resistance to the antiviral protein IFITM2 and enhancement of infection by its paralogue IFITM3 [43]. The increase in resistance could be attributed to one specific mutation in the spike (S) glycoprotein, once again highlighting the importance of surface determinants in modulating the antiviral activity of IFITMs. In contrast to Alpha, the most recent VOC, Omicron, was unique in being sensitive to inhibition by IFITM1, 2, and 3 [44]. Using chimeric spike mutants, the Omicron phenotype’s sensitivity to IFITMs was attributed to the S2 domain of the spike. Omicron emerged later when the population’s humoral immunity was higher. It was the first real neutralizing antibody escape variant, and authors suggested that resistance mutations may have negatively influenced its ability to escape innate immunity, particularly IFITMs’ restriction. This evolution and critical balance between viral evasion of innate and adaptive immunity are similar to what was reported by Foster et al. during the HIV-1 evolution in a host, as mentioned above [37].

While the spike protein appears to modulate the sensitivity of SARS-CoV-2 to IFITM proteins, Kichhoff’s group has shown that experimental design influences the sensitivity of SARS-CoV-2 and other human coronaviruses to IFITM proteins [45,46,47]. Their work suggests that endogenous expression of IFITM2 and/or IFITM3 enhances the replication of both SARS-CoV-1 and SARS-CoV-2, whereas overexpression inhibits them. They also demonstrated that overexpression of IFITM2 and IFITM3 proteins prevents the cell surface expression of ACE2, the entry receptor for SARS-CoV-1 and SARS-CoV-2, thus explaining the inhibitory effect of this artificial overexpression. Furthermore, they showed that the boosting effect of IFITM proteins on viral replication is only observed in the context of native viruses, as pseudoparticles expressing SARS-CoV-1 or SARS-CoV-2 spikes do not reproduce the ability of native viruses to hijack IFITM proteins for efficient infection.

Altogether, these results demonstrate that the antiviral properties of IFITM proteins can be modulated by viral envelope surface glycoproteins. Conversely, beyond merely counteracting the antiviral activity of these proteins, some viruses go to the extent of exploiting them for their own benefit to facilitate replication.

### 2.2. What Are the Mechanisms Involved?

**Figure 2:**
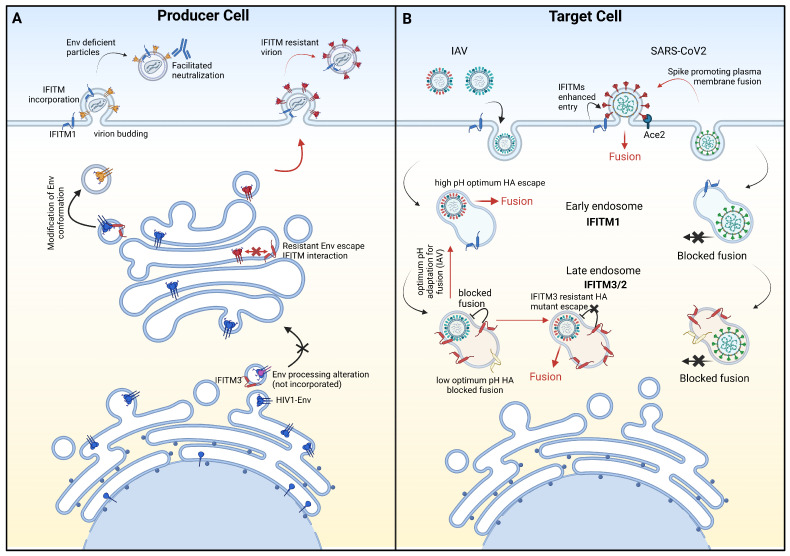
Proposed mechanisms of envelope protein-driven viral resistance to IFITM proteins. The first mechanism of resistance to IFITMs is associated with the loss of interaction between IFITMs and viral envelope proteins. This phenomenon can occur in virion-producing cells (Panel A, illustrated with HIV as an example) and in virus target cells (Panel B, illustrated with IAV as an example). The second resistance mechanism involves reducing the exposure of viruses to IFITMs through modification of their entry pathway. This second mechanism occurs in target cells (Panel B, illustrated with examples of IAV and SARS-CoV-2) (**A**) Representation of the pathway of HIV-1 Env glycoprotein from its synthesis in the endoplasmic reticulum, processing and maturation in the Golgi complex, to its incorporation into viral particles at the plasma membrane. In this pathway, IFITM3 (in red) interacts with the Env of HIV-1 sensitive strains (in blue), resulting in a defect in processing and incorporation into virions or conformational modifications (in yellow) that render the viruses less infectious and more sensitive to neutralizing antibodies. In contrast, the Env of resistant strains (in red) escapes IFITM3 interaction and is incorporated into infectious IFITM3-resistant viral particles. (**B**) Representation of the entry pathways of IAV and SARS-CoV-2. IFITM3-sensitive IAV particles enter target cells through endocytosis, traveling from early to late endosomes, where IFITM3 (in red) interacts with HA2 (in green) to prevent membrane fusion. Conversely, HA2 of resistant strains (in red) escapes IFITM3 through a loss of interaction, allowing membrane fusion in late endosomes (first mechanism) or indirectly through an increase in their optimal pH required for fusion, changing the fusion location from late to early endosomes (second mechanism). The entry of SARS-CoV-2 into target cells is mediated by the spike protein, enabling fusion either at the plasma membrane or in endosomes. When fusion occurs in late endosomes, sensitive spikes (in green) are exposed to IFITM2 (in yellow) and IFITM3 (in red). However, spike adaptation (in red) could favour plasma membrane entry (thus avoiding IFITM2/3), where, under endogenous conditions, IFITMs might act as facilitating co-factors.

While envelope glycoproteins have been shown to modulate restriction by IFITM proteins in several viruses, there is no consensus on the mechanisms involved. Two major non-exclusive mechanisms have been reported (Figure 2). The first would result from a loss or decrease in interaction between IFITMs and viral envelope proteins. The second would involve a reduction in the exposure of viruses to IFITM proteins through modification of their entry pathway.

#### 2.2.1. IFITM Proteins Interact Differentially with Envelope Glycoprotein Variants

HIV-1 Env is synthesized as a single gp160 polyprotein that oligomerizes in the host endoplasmic reticulum into trimers. Trimeric gp160 then transits to the trans-Golgi complex where glycosylation is completed. The gp160 precursor is then cleaved by furin-like proteases into its mature form, composed of gp120, the surface subunit, and gp41, the transmembrane subunit. A pioneer study reported that when virion particles are produced in the presence of IFITM2 or IFITM3, IFITM proteins interacted with HIV-1 Env and impaired its processing and incorporation into virions [33] (Figure 2A). Similar results were reported by Ahi et al. with HIV-based virus-like particles pseudotyped with Env of murine leukaemia virus (MLV), another virus of the *Retroviridae* family [34]. This study also showed that the defective proteolytic processing of Env was due to an IFITM-driven rerouting of Env trafficking to the lysosome for degradation. Similarly, the work of Wrench et al. showed that the isolate specificity of SIV regarding the antiviral activity of IFITM proteins depended on the efficiency of Env incorporation [48].

Nevertheless, several arguments suggest that the downmodulation of Env on the surface of virions is not the only mechanism by which IFITM proteins decrease virion infectivity. Virus strains of different tropism and/or subtypes may differ considerably in their efficiency of processing and incorporation of Env. However, this parameter did not correlate with either their infectivity or their sensitivity to IFITM proteins [35]. Furthermore, the processing defect induced by IFITM proteins remains a matter of debate, as we and others observed no change in the amount of Env incorporated into virions in the presence of IFITM proteins [26,27,35]. Lastly, quantitative comparisons of HIV-1 Env expressed at various concentrations in the presence or absence of IFITM proteins revealed that the level of virion-associated Env did not fully explain the loss of infectivity caused by IFITMs [34]. This suggests that IFITMs also reduced the fusogenic capacity of virions made with Env that remains expressed at their surface [34]. In favor of this hypothesis, we recently confirmed that IFITM3 interacted with Env of IFITM3-sensitive viruses in virus-producing cells, from its synthesis in the endoplasmic reticulum and during its transport to the Golgi complex, right up to its arrival on the plasma membrane at viral particle assembly sites [35]. In addition, using a panel of neutralizing monoclonal antibodies targeting various epitopes on Env, we demonstrated that the incorporation of IFITM3 into virions induced conformational changes of Env that could reduce viral fusogenicity and infectivity (Figure 2 panel A). However, we interestingly observed a loss of interaction of IFITM3 with the Env of IFITM3-resistant viruses, once Env has been processed in the Golgi and is expressed on the cell surface [35]. This suggested that posttranslational modifications underwent by the Env trimer of viruses resistant to IFITM3 would block the access of IFITM3 to its binding site, rendering the Env trimer functional.

Another proposed mechanism of IFITMs’ inhibitory effect could be due to the rigidification of membranes imposed by IFITM proteins [49]. The clustering of Env trimers on virions has been shown to be essential for efficient membrane fusion to occur [50,51]. By rigidifying membranes, Marzialli et al. suggested that IFITMs could interfere with the lateral displacement of Env trimers through the lipid layer, thus hindering clustering and fusion. However, a recent work using super-resolution imaging of Env did not reveal significant effects of IFITMs proteins on Env clustering, suggesting alternative restriction mechanisms [52].

IFITM3 has been described as a potent antiviral protein against influenza A virus (IAV) [53], further supported by the association of an IFITM3 polymorphism with the severity of IAV disease in humans, and increased mortality and morbidity from IAV infection in IFITM3 knockout mice [54,55,56,57]. Nonetheless, distinct patterns of sensitivity to IFITM3 have been described, depending on the viral serotype [12]. Focusing on the inhibitory effect of IFITM3 on viral entry in target cells, a recent study showed that IFITM3 interacted with the membrane fusion subunit of hemagglutinin (HA2) via their transmembrane domain [58]. This interaction occurred in late endosomes and lysosomes and could be a possible mechanism of antiviral action for IFITM3 (Figure 2B). The authors proposed that the susceptibility of different serotypes of IAV to IFITM3 may be related to differences in the affinity of IFITM3 to different HA2. Another study reported that, in addition to exerting its activity in target cells, IFITM3 is incorporated into IAV particles and competes with the incorporation of viral HA [59]. Yet, the mechanism of this competition has not been explored, and the decrease in HA levels in virions had no direct impact on their infectivity. However, interestingly, it sensitized IAV to antibody-mediated neutralization. This latter point will be further discussed below.

Finally, an interaction between IFITM3 overexpressed in target cells and the fusion subunit of the F envelope glycoprotein of Nipah virus (NiV) was recently reported [60]. In contrast to the results discussed earlier, this interaction promoted the NiV envelope-protein mediated entry into target cells.

Similarly, using proximity ligation assays and co-immunoprecipitation analyses, Kichhoff’s group reported that the spike protein of human coronaviruses interacts with the IFITM proteins and exploits them for efficient infection (as mentioned above). However, further analyses of the interaction between IFITM proteins, the spike protein, and the ACE2 receptor at site of viral fusion is required to determine the exact mechanism underlying IFITM-dependent enhancement of infection [45,46].

#### 2.2.2. Envelope Variants Modulate the Pathway of Viral Entry and Exposure to IFITM Proteins

IFITM1 is primarily expressed at the cell surface, whereas IFITM2 and IFITM3 predominantly localize to endosomal compartments [11]. Viral susceptibility to various IFITMs could therefore be modulated by the envelope glycoprotein-dependent entry pathway, depending on whether fusion occurs at the plasma membrane or in endosomes (Figure 2B).

The first evidence of a relationship between susceptibility to restriction and IFITM localization in target cells was reported by Jia et al. regarding IAV [7]. They demonstrated significant enrichment of a minor IFITM3 allele in patients hospitalized for seasonal or 2009 H1N1 pandemic influenza infection. This allele, lacking the amino-terminal 21 amino acids of IFITM3, relocated IFITM3 to the cell surface and was unable to restrict IAV that enters exclusively into target cells through fusion with endosomes. In addition, Gerlach et al. reported that the sensitivity of IAV to IFITM2 and IFITM3 proteins depends on the optimal pH value at which their HA undergoes conformational transitions and mediates membrane fusion. Viruses with a low optimum pH of fusion are more sensitive than viruses fusing at a higher pH [61]. Long et al. proposed that a high optimum pH of fusion would allow IAV to enter the cell from early, rather than late, endosomes [62]. This mechanism may contribute to reducing the susceptibility of the virus to IFITM2 and IFITM3 proteins, primarily localized in late endosomes (Figure 2B).

Regarding HIV, Foster et al. investigated whether the sensitivity to IFITMs’ restriction, dependent on the co-receptor usage of Env, could be linked to the localization of IFITMs [36]. By mutating the binding site for the clathrin adapter AP-2 present on IFITM2/3, resulting in the re-localization of these two proteins to the plasma membrane, R5 viruses became sensitive, whereas restriction of X4 viruses was relieved. This suggests that X4-tropic viruses may preferentially fuse with membranes of different subcellular compartments compared to R5-tropic viruses. Similarly, the higher sensitivity of chronic variants of HIV-1 to IFITM2 and IFITM3, compared to their matched T/F variant, issued from the same individual, was relieved upon the re-localization of IFITM2 and IFITM3 to the plasma membrane [36]. It is widely assumed that HIV-1 enters into target cells through a pH-independent process by fusion of Env and cellular receptors at the plasma membrane. However, the strong inhibition of some variants of HIV-1 by IFITM2 and IFITM3, despite their predominant distribution in endosomes, supports the still controversial hypothesis that HIV-1 could also enter through a functional endosomal pathway [63]. Importantly, under adaptive immune pressure from neutralizing antibodies, chronic variants accumulated escape mutations in Env that were shown to be responsible for this increased susceptibility to IFITM2 and IFITM3. These data argue that amino acids changes in Env under immune pressure impacted the cellular route of viral entry, leading to IFITM2 and IFITM3 restrictions.

Similarly, the restriction of SARS-CoV-2 by IFITM proteins has been reported to be influenced by the viral entry route [43,44,64]. The entry of SARS-CoV-2 into the target cell is initiated by the binding of the viral spike protein to its receptor. To facilitate the fusion of viral and cellular membranes, the spike protein requires a two-step cleavage process. This process begins with an initial cleavage at the S1/S2 junction by furin-like proteases in virus-producing cells [65,66], followed by a second cleavage event at the S2’ site, mediated by TMPRSS2 protease at the surface of the target cell [67]. This allows for viral fusion at the plasma membrane. Alternatively, TMPRSS2-independent cleavage can occur in endosomes by cathepsin proteases, allowing pH-dependent viral fusion with endosomes [65] (Figure 2B). Presence of the furin cleavage site at the S1/S2 junction of the SARS-CoV-2 spike protein has been suggested to play a role in the higher transmissibility of SARS-CoV-2 compared to SARS-CoV-1 [65,68]. Interestingly, Winstone et al. demonstrated that the removal of the furin cleavage site from the spike protein of SARS-CoV-2 restricted viral entry through cathepsin-dependent fusion with endosomes and increased sensitivity to type I interferon and IFITM2 restriction [64]. They proposed that the furin cleavage site enables SARS-CoV-2 to preferentially enter by fusion at the cell surface, thereby reducing its susceptibility to innate immune restriction (Figure 2B). In accordance with this, the work of Lista et al., which reported an increased IFITM resistance of the initial VOC, Alpha, compared to the parental Wuhan-Hu-1 virus as mentioned earlier, has been attributed to a specific amino acid change in the spike adjacent to the furin cleavage site [43]. In the context of the alpha spike, this change further reduced endosomal cathepsin dependence, which is consistent with enhanced cell surface entry. In contrast, the higher susceptibility of the most recent VOC, Omicron, to IFITMs could be explained by its evolution toward an endosomal entry route independent of TMPRSS2 cleavage, exposing it to endosomal IFITMs [69,70]. Put together, these results show that amino acid substitutions in envelope glycoproteins may induce changes in the viral entry pathway and thus indirectly modulate viral susceptibility to IFITMs proteins.

## 3. Interplay between the Innate Restriction by IFITMs and the Humoral Immune Response by Neutralizing Antibodies

The link between innate restriction by IFITMs and the humoral immune response through neutralizing antibodies was first demonstrated by Wrensch et al. for HCV [42]. They provided evidence of a cooperative effect between IFITM proteins and neutralizing antibodies present in sera from infected patients. Using sera at sub-inhibitory concentrations, they demonstrated that IFITM2, overexpressed in target cells, significantly enhances the antiviral activity of neutralizing antibodies. Notably, this cooperative effect was shown to be attenuated by treatment with amphotericin B. Amphotericin B is an amphiphilic antifungal drug that integrates into endosomal membranes, reducing their curvature and increasing fluidity. It can be considered as an IFITM antagonist, acting indirectly by counteracting the rigidification of membranes imposed by IFITM proteins [71]. The loss of cooperation between IFITMs and neutralizing sera after amphotericin B treatment suggested that IFITM-mediated modulation of cellular membranes is responsible for this cooperative antiviral effect.

More recently, another example of viral sensitization to neutralizing antibodies by IFITM proteins has been reported [59]. Lanz et al. demonstrated that the incorporation of IFITM3 into IAV particles competes with the incorporation of viral HA, and although the decrease in HA levels in virions had no direct impact on their infectivity, it made IAV more susceptible to antibody-mediated neutralization [59]. Interestingly, this sensitization effect influenced the infection outcome in an in vivo mouse model of infection. This suggests that IFITM3 may have a significant role in counteracting secondary influenza virus infection when antibodies are present.

In the case of HIV, using neutralizing antibodies targeting various conformational epitopes, we recently reported that, regardless of their sensitivity to IFITM3, viruses that have incorporated IFITM3 had a moderately higher sensitivity to most of these antibodies [35]. Nevertheless, the synergistic effect may not be sufficient, since escape variants to neutralizing antibodies frequently appear in the chronic phase of infection, maybe when the interferon response declines, leading to a reduction in IFITMs levels, thus reducing the pressure exerted on Env.

## 4. Future Directions in IFITM Research

Over the past few years, significant progress has been made in understanding the effects of IFITM proteins on the fusion process of numerous families of enveloped viruses. Initially identified to block virus entry by their presence in target cells, these proteins were later shown to be incorporated into newly budded viral particles, thereby reducing their infectivity. However, it quickly became apparent that certain viruses could escape the antiviral effect of these proteins and in some cases even exploit these proteins to increase their infectivity.

The ability of viruses to escape IFITM restriction was rapidly demonstrated to depend on the nature of their envelope glycoproteins, although the mechanisms of escape remain incompletely understood. While several studies have suggested that IFITM proteins interact with envelope glycoproteins, the mechanistic details of this interaction remain unknown. Similarly, the impact of this interaction on viral infection remains incompletely understood, likely depending on when and where this interaction occurs during the viral infectious cycle and whether it involves other protein partners or not. Although these interactions have generally been demonstrated through co-immunoprecipitation experiments supported by confocal imaging, more in-depth studies are required to demonstrate the direct or indirect nature of these interactions within the infected cell. In this regard, future work could benefit from spatiotemporal imaging techniques such as Förster Resonance Energy Transfer-Fluorescence Lifetime Imaging Microscopy (FRET-FLIM), a sensitive bio-imaging method used to visualize and quantify protein-protein interactions in living cells. However, this technique, while holding several advantages over other methods of protein interaction assays, remains challenging as it requires integrating fluorophore tags into targeted proteins without interfering with their function and localization while maximizing the chance for the tags to be close enough to interact. Mass spectrometry-based methods, such as proximity-dependent biotin identification (BioID) assays, should also be helpful to characterize protein complexes formed between envelope glycoproteins and IFITM proteins.

In addition to modulating their interaction with IFITM proteins, several studies have shown that the nature of envelope glycoproteins can also influence the virus entry pathway, thereby enabling escape from the antiviral activity of these proteins, depending on their localization in cellular membranes. However, these studies have also highlighted the need to be careful regarding the experimental protocols used, as the overexpression of IFITM proteins can artificially modify their effect by indirectly affecting the membrane localization of other cellular proteins, including entry receptors. For example, by impairing the cell surface expression of ACE2, the cell surface receptor of SARS-CoV-1 and SARS-CoV-2, overexpression of IFITM proteins inhibits their infection, while endogenous concentrations of IFITM proteins might favor their infection [45]. In the pursuit of a better understanding of the mechanisms at play in these situations, it would be preferable to specifically focus on experimental setups using depleted cell lines, thereby ensuring the endogenous expression of IFITM proteins.

If viral envelope glycoproteins determine the sensitivity of viruses to IFITM proteins, they also play a crucial role in determining sensitivity or resistance to adaptive humoral immunity, particularly to neutralizing antibodies. Understanding how the interaction between IFITM proteins and envelope proteins could modulate the sensitivity of envelopes to neutralizing antibodies, as suggested for certain viruses, would enhance our comprehension of how IFITM proteins contribute to the broader antiviral response mounted by the cell and their impact on viral evolution.

## Data Availability

Not applicable.
